# Philadelphia Chromosome-Negative Hyperdiploid B-cell Acute Lymphoblastic Leukemia Presenting As Hemophagocytic Lymphohistiocytosis in a Young Adult: A Case Report

**DOI:** 10.7759/cureus.94187

**Published:** 2025-10-09

**Authors:** Abdul Hanan Farooq, Dev Prakash, Ayesha Muneer, Khalid Ahmad, Maha Tariq

**Affiliations:** 1 Acute Medicine, University Hospitals Bristol and Weston NHS Foundation Trust, Weston-super-Mare, GBR; 2 Cardiology, Salford Royal NHS Foundation Trust, Salford, GBR; 3 Medicine, Khyber Medical University, Peshawar, PAK; 4 Surgery, University Hospitals Bristol and Weston NHS Foundation Trust, Bristol, GBR

**Keywords:** b-cell acute lymphoblastic leukemia, hemophagocytic lymphohistiocytosis, hyperdiploid leukemia, malignancy-associated hlh, masked leukemia, philadelphia chromosome-negative b-all, rare clinical presentation

## Abstract

Hemophagocytic lymphohistiocytosis (HLH) is a rare, life-threatening hyperinflammatory syndrome that can mask underlying malignancies. We report a 22-year-old woman who presented with fatigue, night sweats, pancytopenia, hyperferritinemia, and abnormal liver function tests, without peripheral blasts. Imaging revealed no organomegaly or significant lymphadenopathy. Initial evaluation suggested HLH or severe infection. Bone marrow biopsy confirmed Philadelphia chromosome-negative, hyperdiploid B-cell acute lymphoblastic leukemia (B-ALL).

The patient received supportive care with intravenous antibiotics, growth factor support, and tumor lysis prophylaxis. She commenced induction chemotherapy with the UKALL14 Regimen B protocol. Hyperinflammation was controlled with corticosteroids, and she remained clinically stable during induction.

This case highlights the diagnostic challenge of malignancy-associated HLH, particularly when peripheral blasts are absent and imaging is non-specific. Masked leukemia presentations can delay recognition of the underlying cancer, leading to treatment delays. High hyperdiploidy usually confers a favorable prognosis, but its association with HLH is rare.

Clinicians should consider early bone marrow evaluation in young adults with unexplained cytopenias, systemic inflammation, and markedly elevated ferritin, even in the absence of peripheral blasts. Prompt diagnosis and timely initiation of chemotherapy are critical to improve outcomes.

## Introduction

Hemophagocytic lymphohistiocytosis (HLH) is a rare but life-threatening hyperinflammatory syndrome caused by uncontrolled activation of lymphocytes and macrophages, leading to cytokine storm and multiorgan damage [1].

In adults, HLH is most often secondary and may be triggered by infections, autoimmune conditions, or malignancies [2]. Among hematologic malignancies, T- and NK-cell neoplasms are the most frequent causes, but HLH has also been reported rarely in association with B-cell acute lymphoblastic leukemia (B-ALL) [3].

Malignancy-associated HLH (M-HLH) can be a diagnostic challenge because its clinical presentation is characterized by fever, cytopenias, liver dysfunction, and hyperferritinemia, which can mimic sepsis or autoimmune disease and may obscure the underlying cancer [4]. Even in cases of ALL, blasts may be absent from the peripheral blood, delaying recognition of the true diagnosis [5]. HLH can also develop due to immune dysregulation during treatment, highlighting the complex interplay between malignancy, therapy, and hyperinflammation. HLH-like syndromes have also been linked with novel immunotherapies such as chimeric antigen receptor T-cell (CAR-T) in adults with B-ALL [6]. Primary HLH is a genetic disorder presenting mainly in infancy or early childhood due to mutations affecting cytotoxic lymphocyte function.

Here, we report the case of a 22-year-old woman with Philadelphia-negative, hyperdiploid B-ALL who presented with cytopenias, hyperferritinemia, and deranged liver function but no peripheral blasts, closely resembling HLH. This case highlights the importance of maintaining a broad differential diagnosis and the value of early bone marrow evaluation in young patients with unexplained cytopenias and systemic inflammatory features.

## Case presentation

A 22-year-old female of Thai origin, with no significant past medical or drug history, presented with a subacute three-week history of worsening symptoms. She reported progressive fatigue associated with night sweats and a single syncopal episode. She also experienced exertional dyspnea but denied chest pain, dyspnea at rest, rash, oral ulcers, or joint pains. Hair thinning had been noted over the previous five months, which she attributed to academic stress related to recently completing her university finals. Her social history was significant for academic stress while seeking employment. There was no history of recent travel. She was adopted but reported a maternal family history of thalassemia.

**Figure 1:**
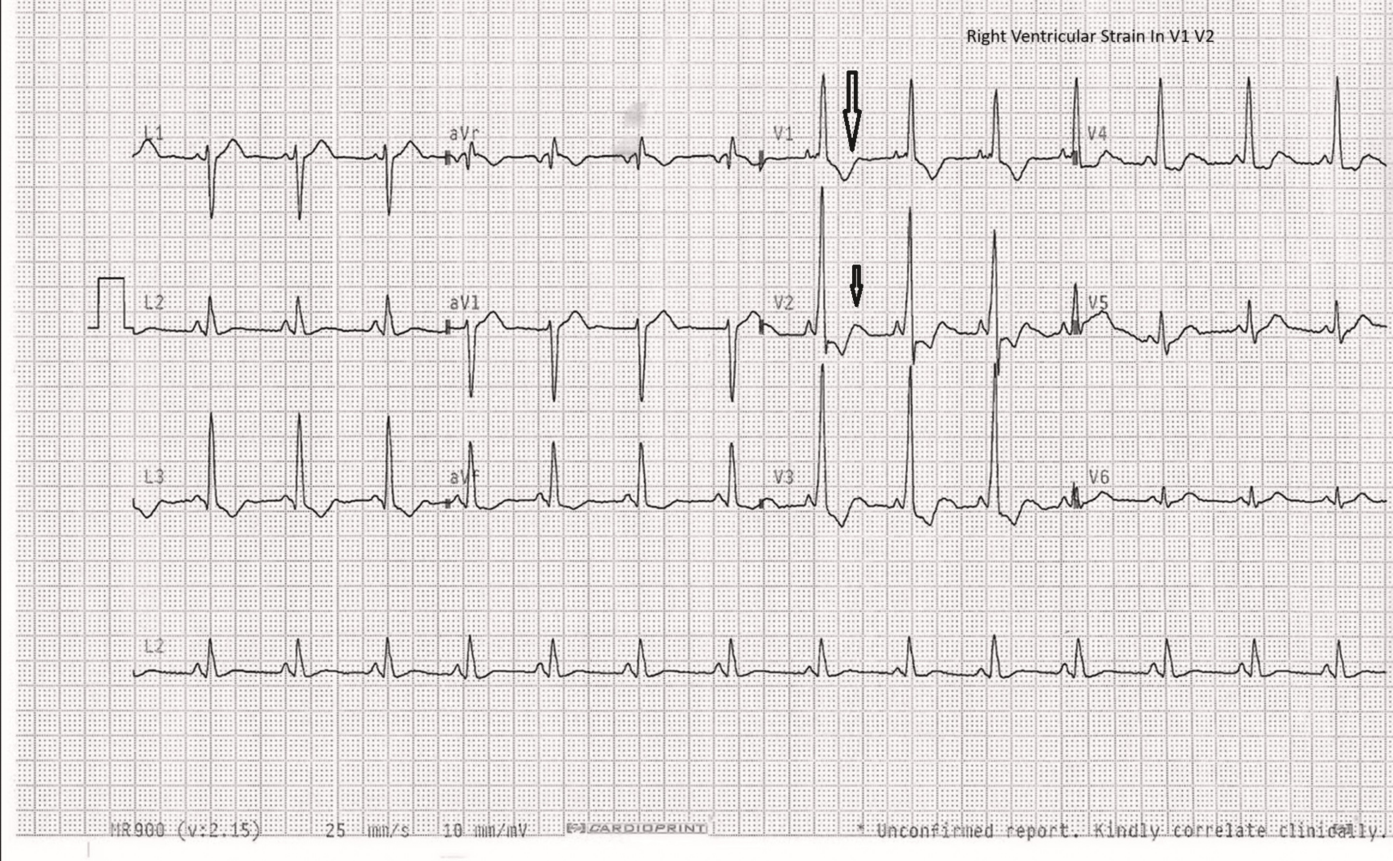
Electrocardiography showing right ventricular strain pattern in leads V1-V2 (arrows)

On admission, her observations revealed mild tachycardia at 102 beats per minute with otherwise stable hemodynamics. Electrocardiography showed right ventricular strain in leads V1-V2 (Figure 1). On examination, there was firm, non-mobile right-sided cervical lymphadenopathy, with no axillary or inguinal lymphadenopathy.

Initial laboratory investigations revealed pancytopenia, with white cell count 0.9 × 10^9^/L, neutrophils 0.31 × 10^9^/L, lymphocytes 0.55 × 10^9^/L, and hemoglobin 10.3 g/dL. The mean corpuscular volume (MCV) was 74.5 fL, and ferritin was markedly elevated at 11,927 μg/L (Table 1). Viral serology confirmed past exposure to cytomegalovirus (CMV) and Epstein-Barr virus (EBV) without evidence of acute infection. The blood film showed hypochromic, microcytic red cells with teardrop cells, pencil cells, and occasional target cells. Severe neutropenia, lymphopenia, and monocytopenia were present. Reactive lymphocytes were noted, but no blasts were seen. Evidence of iron deficiency was present. No evidence of hemolysis was identified. Virology, including CMV and EBV, was suggested.

**Table 1:** Initial laboratory investigations ALT: alanine aminotransferase; AST: aspartate aminotransferase; GGT: gamma-glutamyl transferase; LDH: lactate dehydrogenase; MCV: mean corpuscular volume; MCH: mean corpuscular hemoglobin; ESR: erythrocyte sedimentation rate; RBC: red blood cell

Test	Result	Reference Range
ALT	244 U/L	10-40
AST	356 U/L	<35
Albumin	29 g/L	35-50
Calcium	1.97 mmol/L	2.20-2.60
Adjusted Calcium	2.15 mmol/L	2.20-2.60
D-dimers	18887 ng/mL	≤500
ESR	21 mm/h	≤12
White Cell Count	0.90 ×10^9^/L	4.0-11.0
RBC	3.31 ×10^12^/L	3.80-5.30
Hemoglobin	103 g/L	120-150
Hematocrit	0.248 L/L	0.37-0.45
MCV	75.0 fL	83-100
MCH	25.4 pg	27.0-32.0
Neutrophils	0.31 ×10^9^/L	1.5-8.0
Lymphocytes	0.56 ×10^9^/L	1.0-4.0
Monocytes	0.03 ×10^9^/L	0.2-1.0
GGT	66 U/L	<38
LDH	854 U/L	<250
Triglycerides	5.0 mmol/L	0.5-1.70

Repeat hematology confirmed worsening cytopenias with persistent anemia and neutropenia (Table 2). The direct antiglobulin test was positive for IgG (Table 3).

**Table 2:** Repeat hematology results MCV: mean corpuscular volume; MCH: mean corpuscular hemoglobin; ALT: alanine aminotransferase

Test	Result	Reference Range
White Cell Count	0.90 ×10^9^/L	4.0-11.0
Hemoglobin	84 g/L	120-150
Hematocrit	0.311 L/L	0.37-0.45
MCV	74.9 fL	83-100
MCH	24.9 pg	27.0-32.0
Neutrophils	0.31 ×10^9^/L	1.5-8.0
Lymphocytes	0.55 ×10^9^/L	1.0-4.0
Monocytes	0.03 ×10^9^/L	0.2-1.0
ALT	308 U/L	10-40
Globulin	43 g/L	-

**Table 3:** Direct antiglobulin test (DAT) IgG: immunoglobulin G; C3d: complement component 3d

Test	Result
DAT Screen	Positive (1+)
Anti-IgG	Positive (1+)
Anti-C3d	Negative
DAT Control	Negative

**Figure 2:**
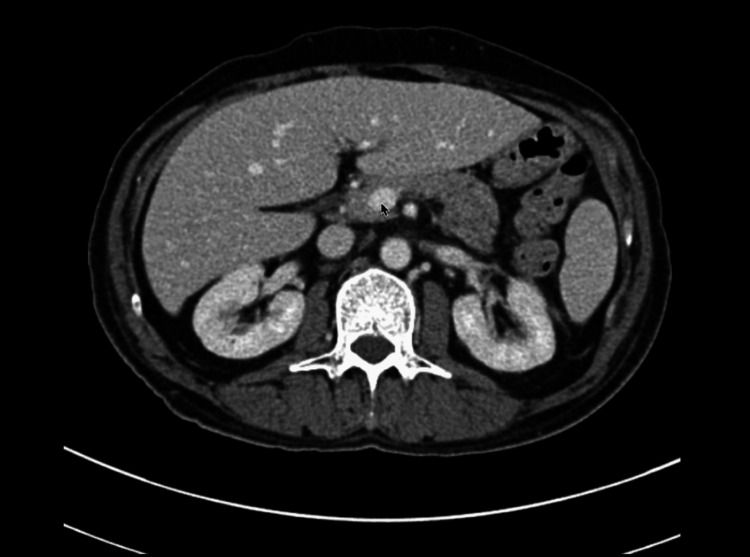
Axial contrast-enhanced CT abdomen showing no hepatosplenomegaly or lymphadenopathy

Cross-sectional imaging with CT head, neck, chest, abdomen, and pelvis did not reveal significant lymphadenopathy or organomegaly, and no focal hepatic lesions or bone abnormalities were seen (Figure 2).

Differential diagnoses at this stage included viral infection, hemophagocytic lymphohistiocytosis (HLH), and hematological malignancy. Given the severity of her neutropenia, she was treated with intravenous Tazocin for neutropenic sepsis and received filgrastim support.

Bone marrow biopsy was subsequently performed, which confirmed a diagnosis of Philadelphia chromosome-negative B-ALL. Cytogenetic analysis revealed high hyperdiploidy, with gains of chromosomes X, 4, 6, 10, 14, 18, and 21, alongside ETV6 loss on copy number array. Fluorescence in situ hybridization (FISH) analysis was normal, and RNA fusion studies were pending at the time. Following stabilization, she was transferred to the regional hematology center, where she commenced induction chemotherapy according to the UKALL14 Regimen B protocol. During induction, she received dexamethasone for four weeks, followed by weaning, allopurinol for tumor lysis prophylaxis, caspofungin, aciclovir, and co-trimoxazole twice daily on weekends for pneumocystis prophylaxis. She was also given low-molecular-weight heparin (LMWH) after each dose of polyethylene glycol-conjugated (PEG) asparaginase for thromboprophylaxis and ciprofloxacin when neutrophil counts fell below 1 × 10^9^/L. Bone marrow restaging was planned at four weeks following induction chemotherapy, with genotyping for further risk stratification.

**Figure 3:**
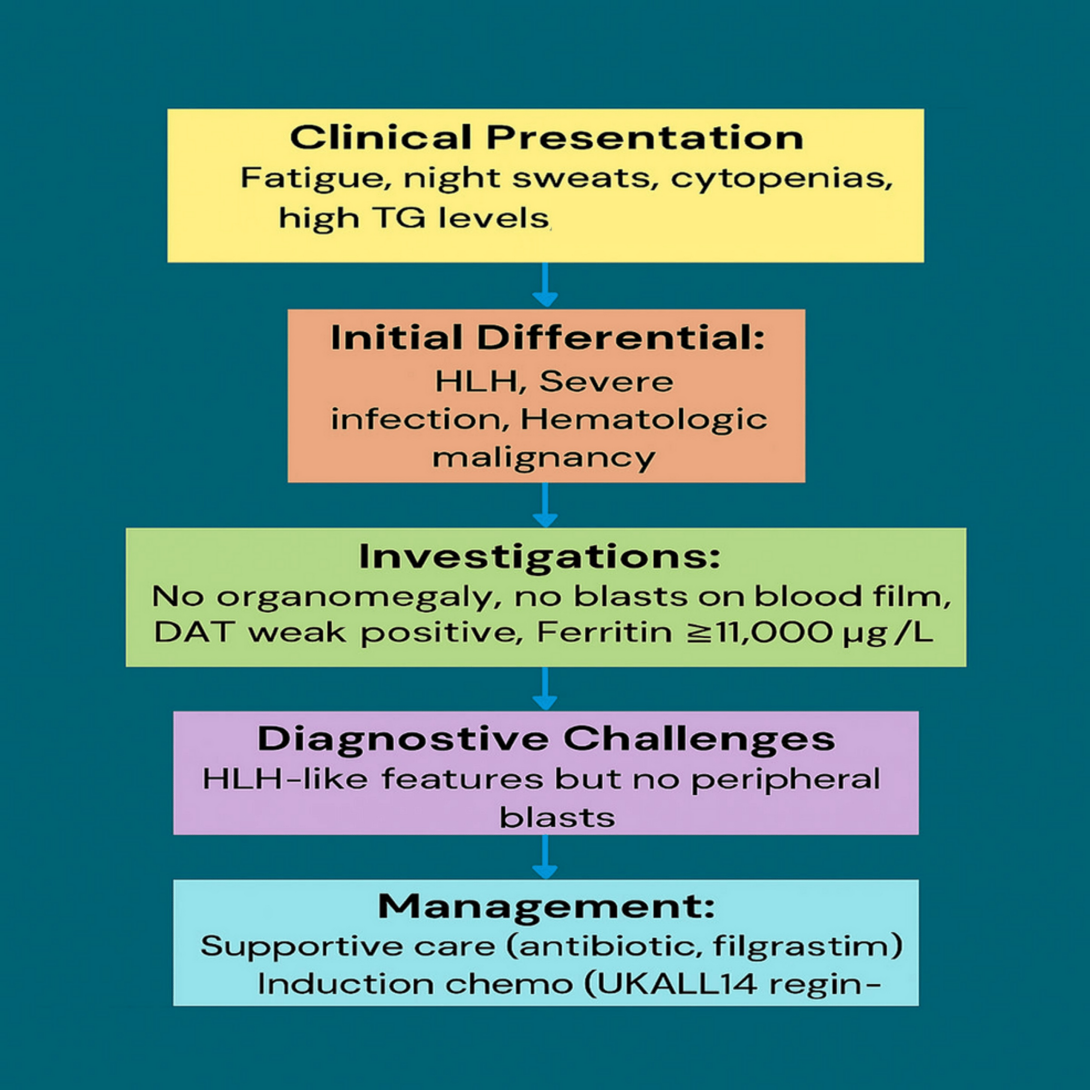
Flow diagram of diagnosis and treatment HLH: hemophagocytic lymphohistiocytosis; TG: triglycerides; DAT: direct antiglobulin test; Ph: Philadelphia chromosome

The diagnostic process and treatment plan are summarized in Figure 3.

## Discussion

M-HLH is a rare but potentially fatal complication of hematologic malignancies, characterized by uncontrolled activation of lymphocytes and macrophages, resulting in a cytokine storm, systemic inflammation, and multiorgan dysfunction [1]. Clinical features such as fever, cytopenias, hyperferritinemia, and liver dysfunction can mimic severe infection or primary HLH, often delaying recognition of an underlying malignancy [7]. Adult patients with B-ALL presenting with HLH pose a particular diagnostic challenge, especially when peripheral blasts are absent [3].

In our patient, pancytopenia, markedly elevated ferritin, abnormal liver function tests, and absent peripheral blasts initially suggested HLH or severe infection. The absence of circulating blasts despite significant cytopenias represents a “masked leukemia” presentation [5]. Imaging showed no organomegaly or significant lymphadenopathy, further complicating the diagnosis. Bone marrow biopsy confirmed Philadelphia-negative, hyperdiploid B-ALL. Although high hyperdiploidy is typically associated with a favorable prognosis, its presentation with HLH is extremely rare [7].

Other reports describe similar diagnostic difficulties. A 57-year-old woman with newly diagnosed Philadelphia-positive, CD20+ B-ALL presented with HLH, showing fever, pancytopenia, hepatomegaly, and hyperferritinemia. Her case highlighted the need for rapid recognition and treatment of both HLH and leukemia, but the outcome remained poor due to disease severity [8]. Similarly, a 57-year-old African American female with Philadelphia-positive, CD20+ B-ALL developed secondary HLH [9]. These reports, together with our case, emphasize that HLH can occur across different B-ALL subtypes and cytogenetic backgrounds, and often predicts a more complex clinical course.

In our patient, no acute viral infection was identified, suggesting HLH was driven primarily by the underlying malignancy. Supportive care with broad-spectrum antibiotics and filgrastim was initiated for severe neutropenia, followed by induction chemotherapy according to the UKALL14 Regimen B protocol. Managing hyperinflammation while treating the underlying malignancy is critical in M-HLH [1,4]. In young adults with unexplained cytopenias and systemic inflammation, a high suspicion for hematologic malignancy allows early diagnosis and prompt treatment. Early bone marrow evaluation, timely therapy initiation, and supportive care are essential to improve prognosis. Recognizing HLH as a presenting feature of B-ALL facilitates rapid symptom management and can improve outcomes [7-9].

## Conclusions

Adult B-ALL mimicking HLH is a rare clinical presentation. The absence of peripheral blasts and the presence of severe cytopenias, hyperferritinemia, and liver dysfunction can make initial recognition difficult. Maintaining a broad differential diagnosis in young adults with unexplained systemic inflammation and cytopenias with early bone marrow evaluation can be crucial in establishing the correct diagnosis, allowing timely initiation of induction chemotherapy and appropriate supportive care. Clinicians should be aware that HLH-like features can precede or coincide with leukemia, and prompt recognition of this rare presentation can prevent delays in treatment and improve patient outcomes.
